# Complications after surgery in patients with colorectal cancer: the evidence for nursing care

**DOI:** 10.1186/1753-6561-5-S6-P196

**Published:** 2011-06-29

**Authors:** JD Silva, HM Sonobe, DD Andrade, AT Giordani, CM Naka Shimura, E Watanabe, NEPECISS

**Affiliations:** 1 General and Specialized Nursing, University of São Paulo, Ribeirão Preto, Brazil

## Introduction / objectives

Patients with colorectal cancer undergoing surgical treatment require planning and preparation of interventions for prevention of postoperative complications, especially considering the complexity and commitment of its clinical and psychosocial condition.

## Objectives

To identify and synthesize the factors that influence the occurrence of postoperative complications and establish the implications of these scientific evidence for nursing care.

## Methods

It is an integrative review of literature where descriptors were used, wound, colorectal cancer and complications with search in the databases Medline, CINAHL and Lilacs, resulting in a sample of 10 scientific articles.

## Results

Results indicated that the preoperative bowel preparation, staging and tumor location, surgical technique and care of the wound in the postoperative period as the factors influencing the occurrence of postoperative complications.

## Conclusion

Thus, measures of prevention and infection control can be implemented that are related to the rigor of the completion of the enema, ensure the conduct, preparation and guidance of patients for diagnostic tests and specialized (CT, MRI and colonoscopy) , fitness and education on pre-operative surgery and its consequences, evaluation of patient outcome and the surgical wound and hospital discharge planning with primary care for the patient and family, encouraging the physiological recovery and wound healing. These nursing interventions could help in decreasing the rates of postoperative complications and mortality of this clientele, as well as improve the quality of perioperative nursing care.

## Disclosure of interest

J. Silva Other review, H. Sonobe: None declared, D. Andrade: None declared, A. Giordani: None declared, C. Naka Shimura: None declared, E. Watanabe: None declared.

